# Feasibility of Automated Segmentation of Pigmented Choroidal Lesions in OCT Data With Deep Learning

**DOI:** 10.1167/tvst.11.9.25

**Published:** 2022-09-26

**Authors:** Philippe Valmaggia, Philipp Friedli, Beat Hörmann, Pascal Kaiser, Hendrik P. N. Scholl, Philippe C. Cattin, Robin Sandkühler, Peter M. Maloca

**Affiliations:** 1 Department of Biomedical Engineering, University of Basel, Allschwil, Switzerland; 2 Institute of Molecular and Clinical Ophthalmology Basel (IOB), Basel, Switzerland; 3 Department of Ophthalmology, University Hospital Basel, Basel, Switzerland; 4 Supercomputing Systems AG, Zürich, Switzerland; 5 Moorfields Eye Hospital NHS Foundation Trust, London, EC1V 2PD, UK

**Keywords:** pigmented choroidal lesion, segmentation, MD-GRU, V-Net, nnU-Net, deep learning, optical coherence tomography

## Abstract

**Purpose:**

To evaluate the feasibility of automated segmentation of pigmented choroidal lesions (PCLs) in optical coherence tomography (OCT) data and compare the performance of different deep neural networks.

**Methods:**

Swept-source OCT image volumes were annotated pixel-wise for PCLs and background. Three deep neural network architectures were applied to the data: the multi-dimensional gated recurrent units (MD-GRU), the V-Net, and the nnU-Net. The nnU-Net was used to compare the performance of two-dimensional (2D) versus three-dimensional (3D) predictions.

**Results:**

A total of 121 OCT volumes were analyzed (100 normal and 21 PCLs). Automated PCL segmentations were successful with all neural networks. The 3D nnU-Net predictions showed the highest recall with a mean of 0.77 ± 0.22 (MD-GRU, 0.60 ± 0.31; V-Net, 0.61 ± 0.25). The 3D nnU-Net predicted PCLs with a Dice coefficient of 0.78 ± 0.13, outperforming MD-GRU (0.62 ± 0.23) and V-Net (0.59 ± 0.24). The smallest distance to the manual annotation was found using 3D nnU-Net with a mean maximum Hausdorff distance of 315 ± 172 µm (MD-GRU, 1542 ± 1169 µm; V-Net, 2408 ± 1060 µm). The 3D nnU-Net showed a superior performance compared with stacked 2D predictions.

**Conclusions:**

The feasibility of automated deep learning segmentation of PCLs was demonstrated in OCT data. The neural network architecture had a relevant impact on PCL predictions.

**Translational Relevance:**

This work serves as proof of concept for segmentations of choroidal pathologies in volumetric OCT data; improvements are conceivable to meet clinical demands for the diagnosis, monitoring, and treatment evaluation of PCLs.

## Introduction

The development of novel deep learning methods has shown substantial improvements for many applications in medical image analysis, such as the automated segmentation of various lesions on the pixel level. The aim of image segmentation is to identify target structures at a pixel level and separate them from the remaining image. In the medical field, image segmentation is used for physiological as well as pathological structures such as tumours.^1^^–^^3^

Optical coherence tomography (OCT) is based on the reflective pattern of laser beams and allows for the imaging of ocular structures at the micrometer scale.^4^ OCT has been used increasingly for the automated segmentation of structures, such as the ocular compartments, retinal layers, or fluids.^5^^–^^7^ Using the vast amount of OCT data, deep learning has facilitated automated segmentation of OCT images at the pixel level for retinal pathologies such as age-related macular degeneration, diabetic retinopathy, and glaucoma.^8^^–^^10^

In contrast to the very abundant deep learning literature concerning the retina, there have been few reports on the choroid.^11^^–^^14^ Pigmented choroidal lesions (PCLs) are a type of choroidal lesion that can be benign (such as nevi) or malignant (such as melanoma).^15^ They are often found incidentally during ophthalmic examination and further characterized using ultrasound examination, fluorescein angiography, or OCT.^16^^,^^17^ OCT is currently not the main imaging modality to investigate PCLs but can be used to quantify their volumes. Its micrometer resolution allows very precise measurements, which could be helpful for PCL monitoring, especially in the case of small lesions.^16^ However, manual annotations for the volumetric quantification of lesions are time-consuming and their quantification could be facilitated by automated segmentation. To the best of our knowledge, there is no report on the automated segmentation of PCLs in OCT data. Therefore, this proof-of-concept study assessed such automated segmentation using three different deep neural networks, named multi-dimensional gated recurrent units (MD-GRU), V-Net, and nnU-Net.^18^^–^^20^

## Methods

### Data Acquisition

Existing data from a previous study were used to compare the segmentation of PCLs.^21^ Thus, the current study was performed in accordance with the ethical standards of the Helsinki Declaration and was approved by the Ethics Committee Nordwest- und Zentralschweiz, Switzerland (ID: EKNZ UBE-15/89 and EKNZ UBE-l5/72). Written informed consent was obtained from all subjects.

All subjects were examined via slit-lamp examination and a clinical fundus inspection before OCT acquisition. Refractive tests without axial length measurements were performed. In the absence of pathological retinal changes, the subject was included in the normal group. The normal eyes were included as a negative control group for the training and testing of the deep learning neural networks. The inclusion criterion for the PCL group was clinical visibility of a melanocytic and relatively well-demarcated lesion that could be recorded entirely with the OCT imaging protocol. There was no exclusion based on the thickness, location, or reflectivity of the PCLs. Exclusion criteria were the absence or inability to consent, presence of trauma, ocular inflammation, poor visibility of the fundus, retinal pathologies such as retinal detachment or subretinal fluid, marked gliosis, presence of a macular hole, vascular occlusion, and inability to steadily fixate.

The data for this study were acquired with a standard swept-source OCT device without dilated pupils (Triton DRI SS-OCT, Topcon, Tokyo, Japan) and comprised optical volumes, each measuring 6 mm × 6 mm × 2.6 mm (256 B-scans, 512 × 992 pixels).

### Manual Annotation of PCLs

**Figure 1.**
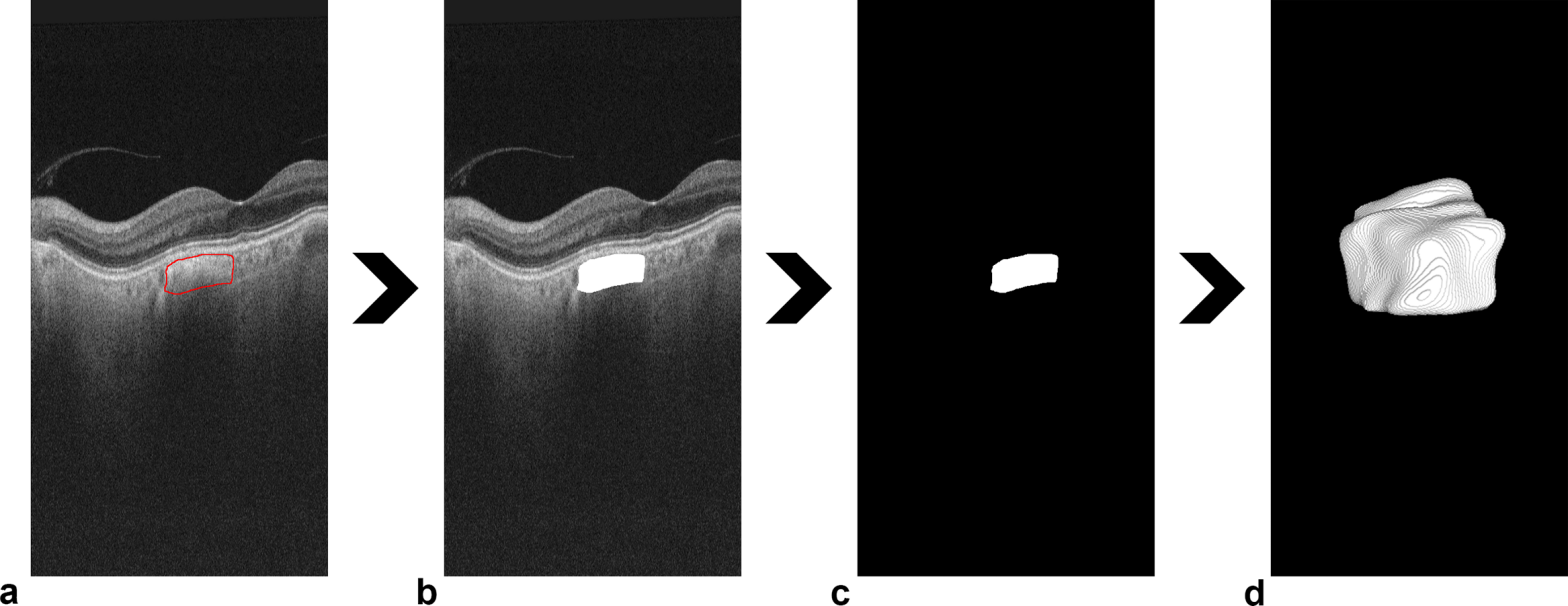
Manual annotation process for PCL segmentation in volumetric OCT data. (a) Outline of the PCL in a single B-scan. (b) Filling of the outline to generate a highlighted PCL. (c) Binary label creation through clearing of the background. (d) Assemblage of the binary labels for each B-scan in the volume produced a 3D label of the PCL.

Two independent human graders were responsible for the manual annotations. PM is a trained ophthalmologist with 25 years of clinical experience and 20 years of experience with OCT systems; PV is a medical student with 2 years of work experience with OCT data. To generate the labels for supervised learning, the OCT volumes were manually annotated with the open source FIJI imaging software v2.1.^22^ The graders visually identified the borders of each PCL using consequential OCT B-scans. The anterior border was delineated at the Bruch's membrane, and the lateral borders were identified in the change of texture between the choroidal tissue and the more homogeneous and hyperreflective PCLs. The posterior border was generated via an interpolation between the choroidal–scleral borders of the adjacent healthy tissue in case the posterior border was not visualizable through the shadow behind the PCL. Finally, each outlined lesion was filled and highlighted with maximum intensity, and the rest of the image was set as a dark background. The manual annotation process is presented in Figure 1.

To test the reproducibility of the method, a test volume consisting of 30 B-scans containing a PCL was annotated three times by each grader; then, the obtained volumes were compared for repeatability using the Dice coefficient. For intragrader reproducibility, the Dice coefficient was calculated between each of the annotations of a single grader (three comparisons per grader); for intergrader reproducibility, the Dice coefficient was calculated between all volumes of different graders (nine comparisons in total).

After stacking the generated annotations, the PCL label was smoothed with 3D Slicer v4.11 (The Slicer Community, Boston, MA, USA), preserving the same dimensions as the original volume.^23^ Hence, for each voxel in the original volume, the corresponding generated label stack indicated the location of the PCL. To optimize the data for neural network training, the volumes were down-sampled to one-half the size in each dimension (128 × 256 × 496 pixels).

### Training of the Neural Networks

Three different neural networks were trained with the data: MD-GRU, V-Net, and nnU-Net. The code for each neural network is publicly available on GitHub.^24^^–^^26^ The MD-GRU was developed at the Department of Biomedical Engineering at the University of Basel.^27^^,^^28^ It was developed specifically for volumetric image segmentation and initially evaluated on tumors as well, namely, for magnetic resonance imaging (MRI)-derived brain data. The V-Net was developed at the Technical University of Munich, also specifically for the purpose of volumetric image segmentation.^19^ The hyperparameters of the MD-GRU and V-Net models were determined empirically on a segmentation dataset containing brain tumours.^2^ The nnU-Net was developed at the German Cancer Research Center to deal with the vast differences found in medical datasets. It provides a U-Net–based neural network that self-configures according to the data it is presented with based on several heuristics, such as the image size, the dataset size, and the available GPU.^20^ nnU-Net was used to compare the performance of two-dimensional (2D) versus three-dimensional (3D) predictions. A summary of the specific neural network configurations is shown in Table 1. Training and testing of the neural networks were performed on an NVIDIA Tesla V100 DGXS 16 GB or a NVIDIA Titan RTX 24 GB (Nvidia, Santa Clara, US).

**Table 1.** Neural Network Configuration Parameters for the 2D and 3D Neural Networks

Neural Network	nnU-Net2D	MD-GRU	V-Net	nnU-Net
Dimensionality	2D	3D	3D	3D
Iterations	250’000	50’000	30’000	250’000
K-folds	5	10	10	5
Batch size	50	1	5	2
Window size	512 × 128	40 × 100 × 50	128 × 128 × 128	64 × 288 × 128
Foreground oversampling (%)	33	50	50	33
Rotation	X	X		X
Mirroring	X	X	X	X
Rescale	X	X		X
Elastic deformation	X	X		X
Gaussian noise	X		X	X
Gaussian blur	X			X
Gamma correction	X			X
Low contrast simulation	X			X
Optimizer	SGD with Nesterov	Adadelta	Adam	SGD with Nesterov
Initial learning rate	0.01	1	0.001	0.01
Descent parameters	Momentum 0.99	Rho 0.9	β1 0.9, β2 0.99	Momentum 0.99

MD-GRU, multi-dimensional gated recurrent units; V-Net, volumetric net; nnU-Net, no-new-net; SGD, stochastic gradient descent.

**Figure 2.**
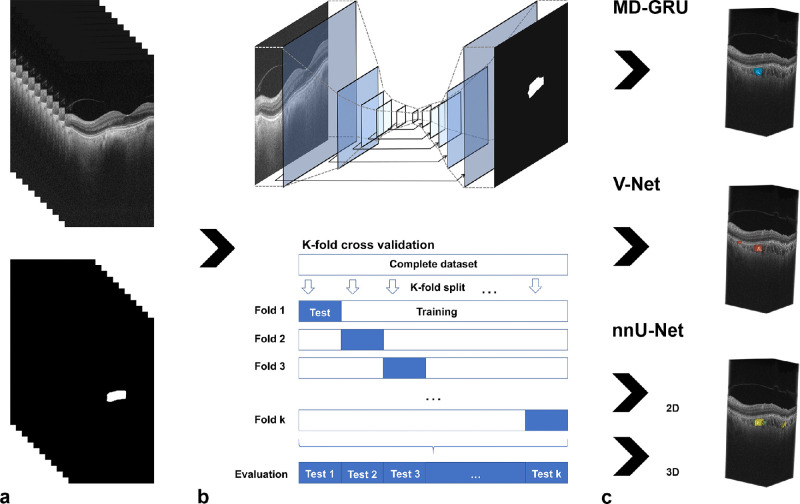
Image processing pipeline for PCL segmentation. (a) OCT data with their corresponding labels were loaded into different deep neural networks (MD-GRU, V-Net, and nnU-Net). (b) Training and testing of the neural networks were performed using k-fold cross-validation with training from scratch for each fold. (c) The resulting lesion predictions are displayed in blue, red, green, and yellow according to each neural network.

The dataset was split into myopic (spherical equivalent <−1.0 diopter [D]), emmetropic (≥−1.0 D and ≤+1.0 D), hyperopic (>+1.0 D), and PCL-containing OCT stacks. These data were then randomly assigned to 10 different equally sized folds. This was done to guarantee that the single folds contained a balanced amount of data from each group, and, in particular, a similar amount of PCL data. Neural networks were then trained and tested with k-fold cross-validation on all folds. For each fold, *k*–1 subsets of the data were used for training, and one was used for testing. The training for each fold was restarted from scratch. The results of all the test subsets were then evaluated as shown in Figure 2b. This practice ensured that none of the training data were used to evaluate the neural network models.

### Data Analyses

The evaluation of the predicted labels was conducted with statistical, similarity, and distance measures using common metrics.^29^ The statistical methods analyzed the parameters according to the confusion matrix between the prediction and the manual annotations. An overview of the formulas used for the evaluation can be found in Supplementary Material 1.

Accuracy was assessed for each neural network. Accuracy is usually a well-established parameter for deep learning; however, in the current study, there was a major class imbalance between the PCL and background voxels. In the present study, a total of 1,966,604,288 matched voxels were present, of which 1,599,136 voxels represented manually annotated PCLs. Hence, if a neural network had predicted all the voxels as background, the accuracy would still have been more than 0.999. This is due to the class imbalance between PCLs and the background data.

A more appropriate evaluation of the neural networks was achieved with the statistical measures recall and precision. Recall is an indicator of the ratio of the relevant data points predicted compared with the total amount in the manual annotation, and precision indicates the ratio of predicted data points that are relevant.^30^ The similarity between the manual annotation and the prediction was assessed with the Dice coefficient. The Hausdorff distance was used to calculate the distance between the two sets, where the maximum and the 95th percentile of the Hausdorff distance are presented. The summarized data are presented as mean ± standard deviation over the cross-validation samples. The programming, configuration of the neural networks, and evaluations were done with Unix shell scripts and Python v.3.6 (Python Software Foundation, Wilmington, DE, USA). The data were visualized with Python v.3.6 and 3DSlicer v4.11.^31^

## Results

In total, 121 labelled OCT volumes from 71 subjects were included in the study. Twenty-one eyes from 21 subjects contributed a PCL, of which 7 were located within the macula. Fifty eyes contributed 100 normal volume OCTs. The normal group consisted of 34 emmetropic eyes, 32 myopic eyes, and 34 hyperopic eyes. The annotations on the test volume showed good reproducibility of the method with a mean intragrader Dice coefficient of 0.92 ± 0.02 and a mean intergrader Dice coefficient of 0.88 ± 0.02.

### Quantitative Analysis

All neural networks generated automated PCL segmentation. The 3D nnU-Net generated the best predictions regarding the mentioned evaluation criteria. The recall of the 3D nnU-Net was 0.77 ± 0.22, and the precision was 0.59 ± 0.38. Regarding the precision, the nnU-Net2D trained on only 2D scans generated the best results at 0.69 ± 0.32. The mean Dice coefficient for the predictions of the 3D nnU-Net was 0.78 ± 0.13, outperforming MD-GRU (0.62 ± 0.23) and V-Net (0.59 ± 0.24). This Dice coefficient indicates that there is a major overlap between the predicted and manually annotated PCLs. Furthermore, the predicted PCLs were of similar size compared with the manual annotations. The 3D version of the nnU-Net performed best in terms of distance metrics, with a maximal Hausdorff distance of 315 ± 172 µm (MD-GRU, 1542 ± 1169 µm; V-Net, 2408 ± 1060 µm). In a comparison of models trained on 2D and 3D, the 3D version of the nnU-Net was superior to the 2D version regarding nearly every metric. A detailed summary of the evaluation parameters for 2D and 3D is presented in Table 2.

**Table 2.** Evaluation Parameters for the 2D and 3D Neural Networks

	nnU-Net2D		MD-GRU		V-Net		nnU-Net	
Neural Network	Mean ± SD	Vol	Mean ± SD	Vol	Mean ± SD	Vol	Mean ± SD	Vol
TP	5749 ± 18427	121	7873 ± 23550	121	7217 ± 20327	121	9087 ± 24846	121
TN	16238846 ± 47561	121	16232177 ± 56644	121	16230628 ± 61766	121	16236763 ± 50671	121
FP	866 ± 2541	121	7535 ± 25007	121	9084 ± 34105	121	2949 ± 11083	121
FN	7467 ± 31243	121	5343 ± 26746	121	5999 ± 30822	121	4129 ± 25702	121
Tot pos label	13216 ± 46305	121	13216 ± 46305	121	13216 ± 46305	121	13216 ± 46305	121
Tot pos prediction	6615 ± 20217	121	15408 ± 38250	121	16301 ± 44519	121	12036 ± 31158	121
Accuracy	0.999 ± 0.002	121	0.999 ± 0.002	121	0.999 ± 0.003	121	>0.999 ± 0.002	121
Recall	0.438 ± 0.276	21	0.599 ± 0.305	21	0.611 ± 0.252	21	0.773 ± 0.222	21
Specificity	>0.999 ± 0.000	121	>0.999 ± 0.002	121	0.999 ± 0.002	121	>0.999 ± 0.001	121
Precision	0.687 ± 0.319	23	0.318 ± 0.385	41	0.134 ± 0.298	102	0.588 ± 0.383	27
NPV	>0.999 ± 0.002	121	>0.999 ± 0.002	121	>0.999 ± 0.002	121	>0.999 ± 0.002	121
FP rate	0 ± 0.000	121	0 ± 0.002	121	0.001 ± 0.002	121	0 ± 0.001	121
FN rate	0.562 ± 0.276	21	0.401 ± 0.305	21	0.389 ± 0.252	21	0.227 ± 0.222	21
False discovery rate	0.313 ± 0.319	23	0.682 ± 0.385	41	0.866 ± 0.298	102	0.412 ± 0.383	27
False omission rate	0 ± 0.002	121	0 ± 0.002	121	0 ± 0.002	121	0 ± 0.002	121
Dice	0.547 ± 0.253	20	0.622 ± 0.230	19	0.593 ± 0.238	20	0.779 ± 0.129	20
HDmax [µm]	1011 ± 892	20	1542 ± 1169	19	2408 ± 1060	21	315 ± 172	20
HD95 [µm]	593 ± 702	20	953 ± 1083	19	1176 ± 1147	21	153 ± 95	20

FN, false negative; FP, false positive; HDmax, maximal Hausdorff distance; HD95, 95th percentile of the Hausdorff distance; NPV, negative predictive value; SD, standard deviation; Tot pos, total number of positives; TP, true positive; TN, true negative; Vol, number of volumes (=eyes).

Values of >0.999 indicate that the rounding with precision of three digits would be 1.

### Qualitative Analysis

**Figure 3.**
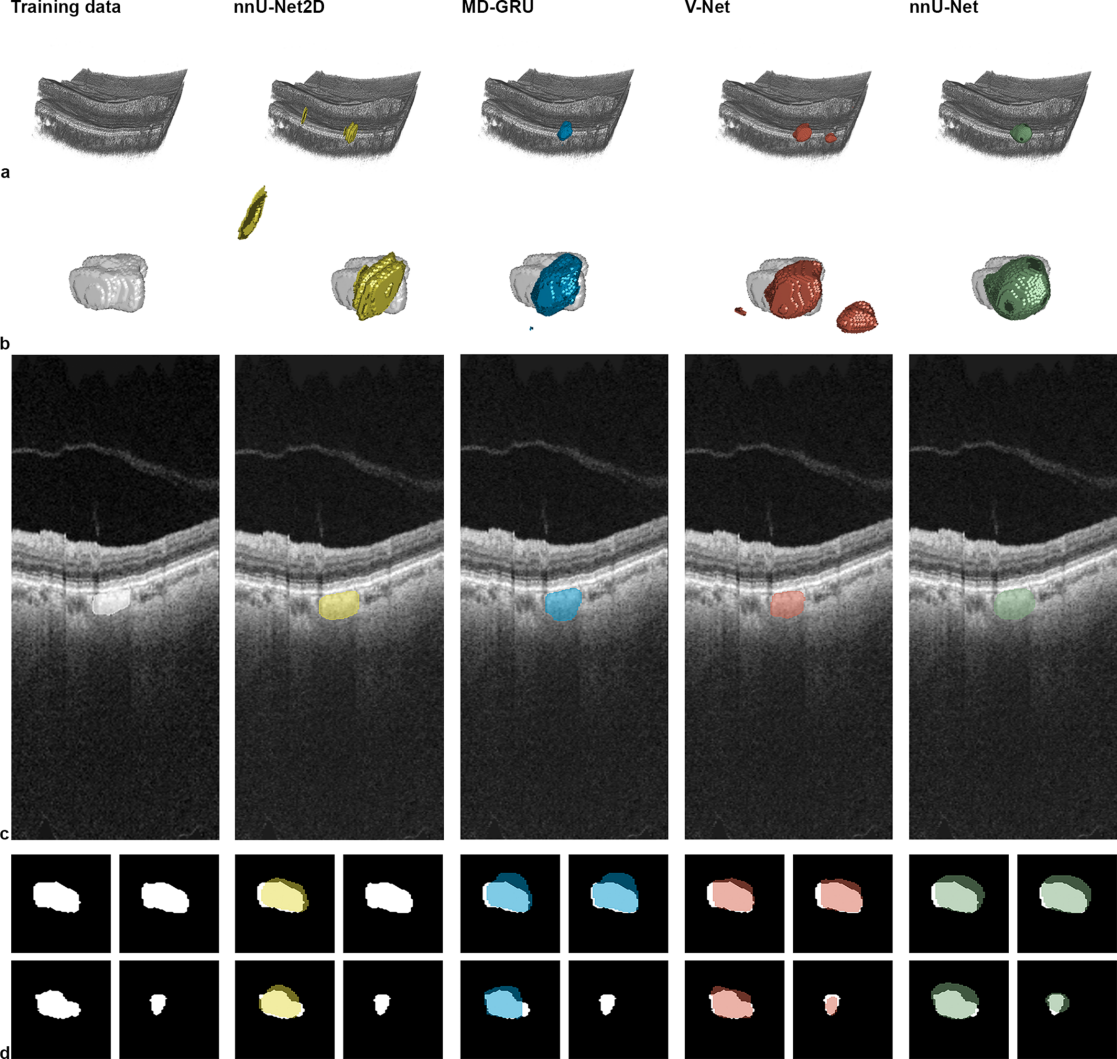
Visualisations of the neural network predictions with overlays on the OCT images and the manual annotations. (a) Volume-rendered retinal and choroidal compartments. (b) Three-dimensional manual annotations and model predictions. (c) Two-dimensional OCT images and predictions as overlays. (d) Enlarged 2D manual annotations and predictions as overlays.

This superior performance of the volumetric neural networks is visualized intuitively in Figure 3a and 3b. The 3D PCL predictions were more coherent than the split predictions for the 2D nnU-Net. On single B-scans, the predictions for all the neural networks were often very similar (Figure 3c). Yet when analyzing the entire C-Scan, nnU-Net2D missed PCLs in single B-scans, even though the lesion was identified in adjacent B-scans. An example of missed lesions in B-scans by the 2D neural network in the PCL core is illustrated in Figure 3d. These missing predictions are due to the input into the neural network where the spatial volume context is lost when only training with isolated 2D images.

**Figure 4.**
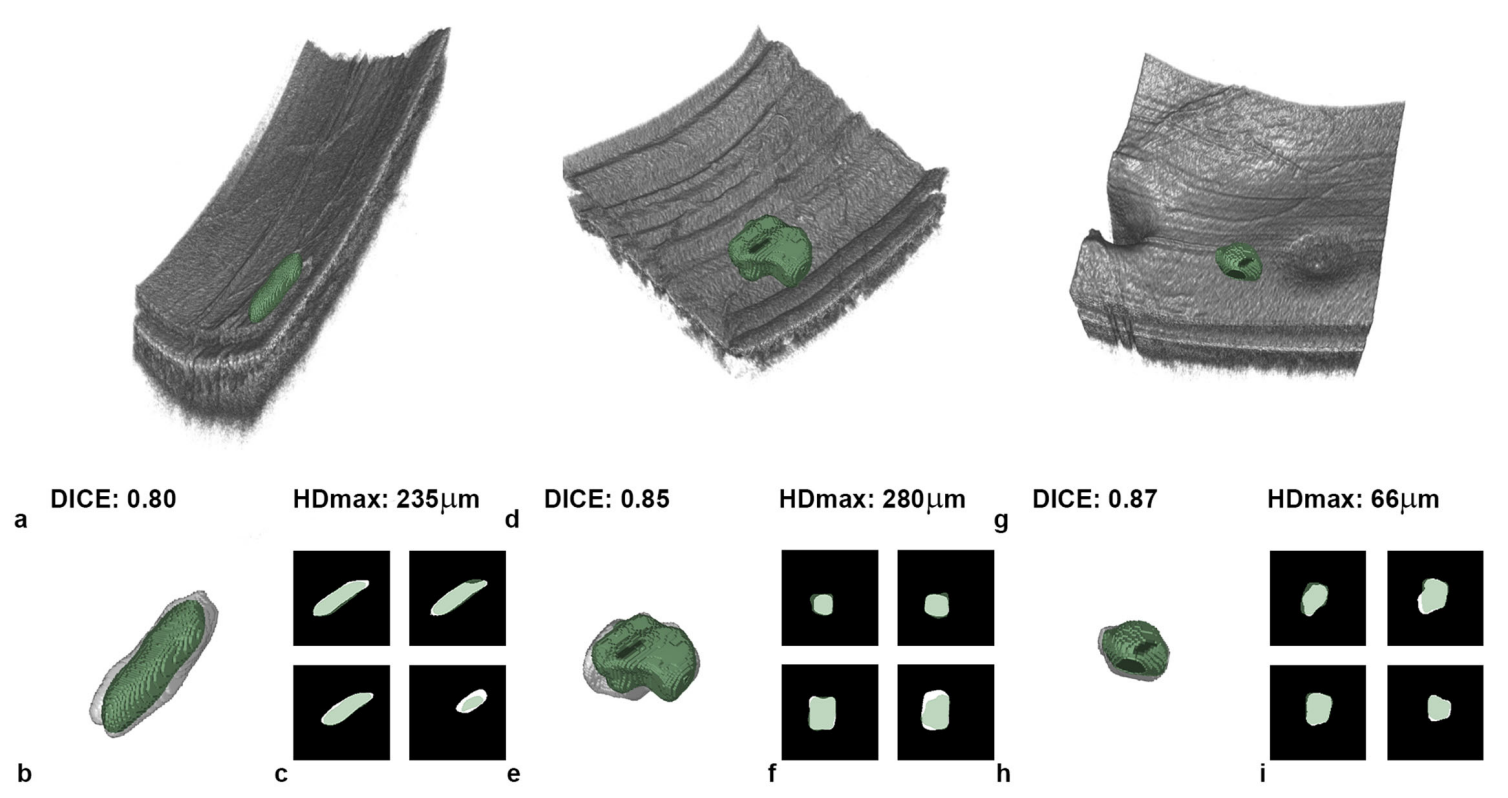
Example automated PCL segmentations generated using 3D nnU-Net. PCL predictions are shown as a green overlay, and manual annotations in are shown in white. (a, d, g) Volume rendered retinal and choroidal compartments. Predicted PCL with corresponding Dice coefficient and maximum Hausdorff distance. (b, e, h) 3D predictions overlaid over the manual annotations. (c, f, i) Enlarged 2D manual annotations with overlaid predictions. Axial stretching was performed for a better visualization according to isometric pixels.

The visualizations provide further insight into the segmentation performance of the evaluated neural networks. Overall, the 3D nnU-Net provided the most condensed segmentations of PCLs (Figure 3). Three additional examples are included in Figure 4, where the generated predictions are presented with the individual Dice coefficient and the maximum Hausdorff distance. As can be seen, the predicted shapes are centrally located in the manual annotation; the borders correspond with the manual annotation, slightly surpassing or missing the border. The data show that different shapes and locations of PCLs can be predicted, be it a peripheral longitudinal PCL (Fig. 4a), a tortuous PCL on the posterior pole (Fig. 4b), or a small PCL between the optic nerve head and the macula (Fig. 4c).

## Discussion

A PCL is common and typically identified as a brownish, darkly pigmented lesion in the course of a routine clinical investigation of the fundus.^32^^,^^33^ Although the majority of PCLs are benign choroidal nevi, malignant transformation is still possible and is highly difficult to detect at an early stage, because tissue sampling for diagnosis is not possible without damaging healthy tissue.^34^^,^^35^ In this context, OCT may represent a possible imaging tool to enable early PCL diagnosis at a micrometer level. It could be used to guide the quantitative monitoring of PCLs and support treatment decisions, particularly if it is enhanced with deep learning tumor segmentation.^36^

This study presents the first successful automated segmentation of PCLs in OCT data. In addition, it compares the segmentation performance of three different deep learning architectures, MD-GRU, V-Net, and nnU-Net. Overall, the nnU-Net trained on 3D data showed the best performance, outperforming the other models on the key metrics of recall, Dice coefficient, and maximum Hausdorff distance.

Recall, also known as sensitivity, punishes large numbers of false negatives in the prediction. Recall was chosen as the key statistical metric, because it is crucial not to miss anomalous findings. If the analyzed PCL in future evaluations was malignant, it would be even more important not to miss parts of the PCL. The best-achieved recall score of 0.77 ± 0.22 was achieved with the 3D nnU-Net, which means that the algorithm detected around three-quarters of all lesion voxels.

Further, the shape and position of the predicted PCL should correspond with the actual underlying tumor. For the similarity evaluation, the Dice coefficient was used, which is a commonly used key metric in image segmentation.^20^ The resulting values of the 3D nnU-Net model with a mean Dice coefficient 0.78 could be used as an orientation when comparing future PCL segmentations. Although the Dice coefficients for different tasks are difficult to compare, the presented model generated predictions in the same similarity range as neural networks in the RETOUCH challenge. This challenge investigated the segmentation of different fluids in retinal OCT images where the best-performing team achieved a mean Dice coefficient of 0.77, and the average of the participating teams was 0.68.^7^^,^^37^ It is also notable that PCL segmentation is thought to be a harder task than retinal fluid segmentation, because the borders are more diffuse for PCLs than retinal fluids in OCT images.

The deviation between the manual annotation and the prediction can be visualized and qualitatively evaluated in 3D as shown in Figure 4. The mismatch can best be quantified with the Hausdorff distance. The Hausdorff distance is a good measure for translating the pixel domain into real-world measures. For example, with the relatively large voxel sizes in clinical MRI, a single-pixel deviation means that a deviation of more than 1 mm would be induced. A small distance between predictions and real localization is particularly important for the radiotherapy guidance of malignant melanoma.^38^ With regard to the OCT pixel domain, a deviation of a single pixel corresponds only to a deviation in the micrometer range. The proposed 3D nnU-Net prediction had an average maximum Hausdorff distance of 315 ± 172 µm.

The Dice coefficient as a sole criterion can be misleading in ocular lesions. As shown in Figure 4, PCLs 4a and 4c had an almost identical Dice coefficient, but the segmentation quality could be further discriminated by the Hausdorff distance in µm. Conversion from pixel distance to micrometers was performed by readjusting to the original voxel sizes. This measure was relevant, as it is possible that the predicted segmentation was correct for a large part of the lesion and had a good score on similarity but omitted a section of the PCL (such as a spur).

The implemented neural network configuration was important; major deviations between the different neural network architectures were found. Although MD-GRU and V-Net were trained with parameters that worked well for segmentation tasks, the self-configuring nnU-Net framework performed best. The nnU-Net is based on a heuristic approach, which determines the neural network configuration based on the input data characteristics and the available hardware.^20^ Because the settings from the heuristic approach might be further optimizable, this shows that using state-of-the-art neural network architectures is relevant for the tasks. Further, in this study, the 3D model outperformed the 2D model, which produced some nonphysiological predictions with gaps on single B-scans. As all corporal structures are volumetric, 3D segmentation models should be selected when feasible.

This work could have translational relevance for patients with PCL because the obtained segmentations allow for a novel kind of quantification of the PCL volume and surface area. Clinically, it is not always evident if such a lesion has a risk of malignant transformation.^15^ Using OCT-derived volume analysis, the evolution of potentially malignant lesions could be followed up quantitatively, and the automated segmentation of PCLs could support clinical decisions. The risk for metastasis could be monitored at a micrometer scale, compared with conventional ocular ultrasound imaging that can only characterize lesions at a millimeter scale at a high enough accuracy.^39^^–^^42^ Therefore, automated segmentation of these small lesions with OCT could potentially enhance follow-up for patients and support an earlier diagnosis of malignant PCL transformation.

Automated segmentation could facilitate the radiotherapy of malignant lesions; for this, the correct demarcation of the target tissue is essential. The image modalities used in a common proton beam radiotherapy planning include fundus photographs, ultrasound examination, orthogonal radiographs, and MRI.^43^^–^^45^ In the future, the researchers assume that OCT imaging could be integrated in adjunction to other modalities into such therapy planning pipelines, because it provides volumetric information and a much higher resolution than other imaging modalities currently in use. Automatically segmented lesions could be highlighted and controlled by a clinician, which could ease or even eradicate the time-consuming process of manual annotations. By integrating automated deep learning segmentation into the data visualization pipeline, the structural understanding of a PCL could be facilitated for both clinicians and patients.

In terms of limitations, the current work has a relatively low number of PCL cases that were not part of a screening population. The control group consisted of healthy participants without PCL masquerades. However, the main goal of this study was to investigate feasibility and compare different deep learning algorithms, which was successful. The obtained data were derived only from one OCT device without enhanced-depth imaging. However, the used swept-source OCT technology allowed the visualization of the choroid and ensured data homogeneity for this initial proof-of-concept study. To investigate the generalizability of automated PCL segmentation, future studies should test for domain adaptation with different acquisition protocols, diverse study populations and different OCT scanners.

The intrasubject evolution of PCLs was not assessed and has yet to be tested to check for the feasibility of a quantitative long-term follow-up study. The quantitative monitoring of the lesions could compare automated segmentation against manual annotations, because, to our knowledge, the volumetric monitoring of PCLs with OCT has not yet been presented. Another limitation was that a histological examination of the PCLs was not possible without harming healthy tissue. Thus, the nature of the lesion could not be determined, and it remained unknown whether the lesions were choroidal nevi or if they were malignant tumors. However, because only relatively small, well-delineated PCLs were included, it can be assumed that the majority were choroidal nevi. Furthermore, human annotation of PCLs may show deviations; future manually annotated data from different graders should be included in studies going beyond the present proof-of-concept study.

Improving the automated segmentation could be achieved by modifying the neural networks. The three different neural networks used here were trained with the best-performing hyperparameters, but they were not further optimized for this task. In further optimized configurations, the class imbalance of the PCL pixel and the background pixel could be taken into account during training for further improvements.^46^ As the self-configuring nnU-Net performed the best, optimization for other network architectures seems feasible. Such improvements and further validation are needed to meet clinical demands for the diagnosis, monitoring, and treatment evaluation of PCLs.

## Conclusions

This proof-of-concept study showed that automated segmentation of PCLs in OCT data with deep learning is feasible. The neural network architecture had a relevant impact on PCL predictions. Overall, the 3D nnU-Net showed the best performance among the evaluated models. Automated deep learning segmentation could be integrated into a volumetric visualization pipeline and facilitate structural understanding for both clinicians and patients.

## Supplementary Material

Supplement 1
