# Supplementary material for: Feasibility of Automated Segmentation of Pigmented Choroidal Lesions in OCT Data With Deep Learning
Source: Transl Vis Sci Technol. 2022 Sep 26;11(9):25. doi: 10.1167/tvst.11.9.25 (PMC9526362; doi:10.1167/tvst.11.9.25)
Supplement: Supplement 1 [file tvst-11-9-25_s001.pdf]

Supplementary Material 1. Evaluation parameters

A. Statistical measures

Four cardinal measures

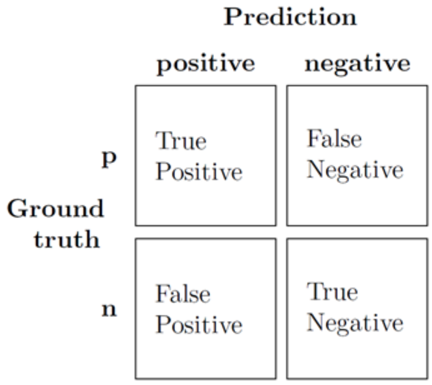

True positives (in voxel) *TP*

True negatives (in voxel) *TN*

False positives (in voxel) *FP*

False negatives (in voxel) *FN*

Number of positive data points

Total positives in the manual annotation

Total positives in the prediction

Accuracy

The accuracy measures the rate of correctly identified data points.

$$\frac{TP + TN}{TP + FP + TN + FN}$$

Recall

The recall is also called the 'sensitivity' or 'true positive rate'.

$$\frac{TP}{TP + FN}$$

Specificity

The specificity is also called the 'true negative rate'.

$$\frac{TN}{TN + FP}$$

Precision

The precision is also called the ‘positive predictive value’.

$$\frac{TP}{TP + FP}$$

Negative predictive value

$$\frac{TN}{TN + FN}$$

False positive rate

$$\frac{FP}{FP + TN}$$

False negative rate

$$\frac{FN}{FN + TP}$$

False discovery rate

$$\frac{FP}{FP + TP}$$

False omission rate

$$\frac{FN}{FN + TN}$$

B. Similarity measures

DICE coefficient

The DICE coefficient corresponds to the F1 score.

$$DICE = \frac{2 * Precision * Recall}{Precision + Recall}$$

or

$$DICE = \frac{2 * TP}{2 * TP + FP + FN}$$

C. Distance measures

Hausdorff distance

The Hausdorff distance *h(A,B)* is defined as the maximum distance d of a point a in set A to the nearest point b in the other set B and can be computed as a *maxmin* function for the sets A and B.

$$h(A,B) = \max_{a \in A} \{\min_{b \in B} d(a,b)\}$$

As the Hausdorff distance is not symmetric, generally, *h(A,B)* is not equal to *h(B,A)*. For this work, a more general definition of the Hausdorff distance *H(A,B)* was used.

$$H(A,B) = \max\{h(A,B), h(B,A)\}$$

For the Hausdorff distances, the distance from each point in one set is calculated to each point in the other set. To allow an interpretation without the impact of major outliers, the 95th percentile of the distances between both sets is also presented in the manuscript.
